# Supplementary figures and images for: Kindia (Pavetteae, Rubiaceae), a new cliff-dwelling genus with chemically profiled colleter exudate from Mt Gangan, Republic of Guinea
Source: PeerJ. 2018 Apr 20;6:e4666. doi: 10.7717/peerj.4666 (PMC5912204; doi:10.7717/peerj.4666)

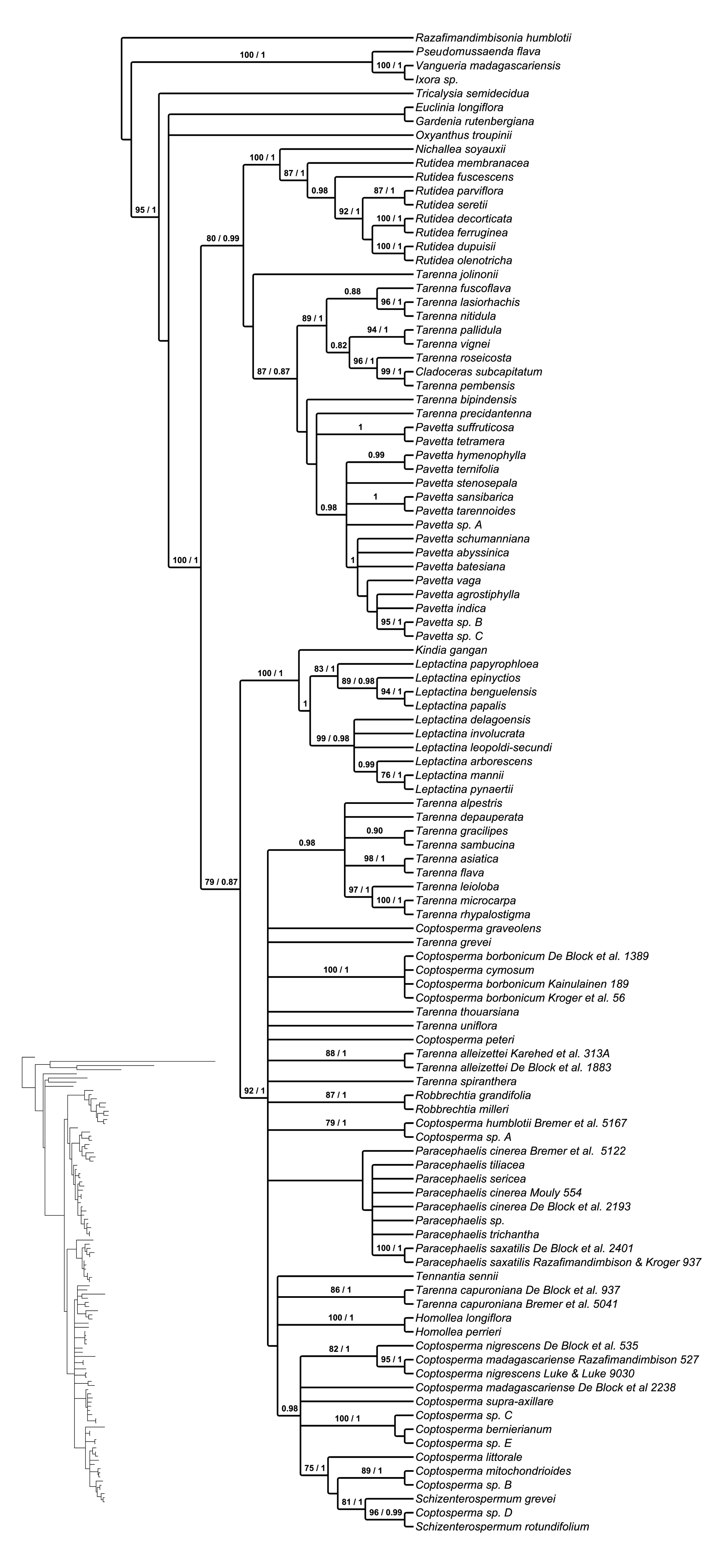

Supplement: Figure S1 — Only PP above 0.80 and BS values above 75% are shown. Nodes with PP < 0.5 support have been collapsed. Inset tree shows the branch lengths. [file peerj-06-4666-s002.png]
